# improving Pain mAnagement for childreN and young people attendeD by Ambulance (PANDA): protocol for a realist evaluation and consensus workshops.

**DOI:** 10.3310/nihropenres.14025.1

**Published:** 2025-10-01

**Authors:** Gregory Adam Whitley, Georgie Nicholls, Michael Baliousis, Tatiana Bujor, Jessica Hodgson, Paul Leighton, Bill Lord, Sarah Redsell, Kacper Sumera, Aloysius Niroshan Siriwardena

**Affiliations:** 1 Clinical Audit and Research Unit, East Midlands Ambulance Service NHS Trust, Lincoln, UK; 2 Community and Health Research Unit, University of Lincoln, Lincoln, UK; 3 School of Psychology, University of Lincoln, Lincoln, UK; 4 Nottingham University Hospitals NHS Trust, Nottingham, UK; 5 School of Medicine, University of Nottingham, Nottingham, UK; 6 School of Medicine, Newcastle University, Newcastle upon Tyne, UK; 7 Applied Health Research Building (Building 42), University of Nottingham, Nottingham, UK; 8 Monash University, Clayton, Victoria, Australia; 9 Faculty of Medicine & Health Sciences, University of Nottingham, Nottingham, UK; 10 East Midlands Ambulance Service NHS Trust, Nottingham, UK

**Keywords:** Acute Pain, Analgesia, Child, Emergency Medical Services, Paramedics, Paediatrics.

## Abstract

**Background:**

Prehospital pain management in children and young people (CYP) is a top research priority in paediatric emergency medicine. Approximately 90,000 CYP under 18 years of age are transported to hospital by ambulance each year for conditions associated with acute pain in England. Approximately half of the children suffering from acute pain do not receive analgesics from paramedics, and approximately 60% do not experience a meaningful reduction in their pain severity. The aim of these studies is to explore the experiences of key stakeholders, refine our programme theory, and prioritise candidate intervention components to address the gaps in care.

**Methods:**

A realist evaluation using a multiple case study approach following the RAMESIS II guidance and consensus workshops using the modified nominal group technique will be performed. The realist evaluation will collect qualitative data (interviews, diaries, electronic messaging, arts-based materials) from CYP aged 4 – 17 years who have experienced acute pain and needed an ambulance in the previous 12 months, parents and carers, and ambulance clinicians. Recruitment will occur across England within three NHS ambulance services, two NHS children’s emergency departments, and via social media. We will also collect routine quantitative clinical record data from two NHS ambulance services. We will use a realist logic of analysis to code the data into conceptual buckets, develop context-mechanism-outcome configurations, and refine the programme theory developed from a previous realist review. Two consensus workshops will be held: one for CYP aged 8-17 years, parents and carers, public representatives, and members of the Young Persons Advisory Group, and one for ambulance clinicians, quality leads, and educators. Workshop attendees will review the study findings and candidate intervention components, vote on their importance, and review voting results. High-priority intervention components will be considered for incorporation into an intervention.

## Plain Language Summary

**Background:**

All children and young people (CYP) experience pain at some point in their lives. While pain is inevitable, suffering is not. Approximately 90,000 CYP under 18 years of age are transported to hospital by ambulance each year for conditions associated with acute pain in England. Many of these patients do not receive pain relief medication from paramedics or have their pain sufficiently reduced to make a difference. Not managing pain well in the ambulance can have a lasting impact on a child’s physical and mental well-being. The aims of these studies are to explore the experience of CYP, parents and carers, and ambulance clinicians, update an existing theory about what works well for CYP, and rank a list of pain management improvement methods.

**Methods:**

In the first study, CYP aged 4 to 17 years, parents, carers, and ambulance clinicians will be invited to share their experience of ambulance care. This may be via interviews, diaries, electronic messaging, or the use of arts and crafts. Recruitment will occur across England within three ambulance services and two children’s emergency departments and online via social media and website advertising. We will also analyse the clinical records of CYP who needed an ambulance for a painful condition from two ambulance service. These data will allow us to build a theory on what works well for different children and young people. In the second study, we will use this theory to develop a list of pain management improvement methods. This list of methods will be reviewed by CYP, parents and carers, ambulance clinicians, quality leads, and educators to determine which are the most important and most likely to make a difference. We will then use this list to create a new way to improve pain management for CYP who need an ambulance.

## Introduction

Prehospital pain management has been identified as a top research priority in paediatric emergency medicine ^1, 2^ and a key outcome measure for ambulance services ^3^. A recent call to arms to “make children’s pain matter” by Eccleston *et al*. ^4^ has further placed pain management in children and young people (CYP) under the spotlight, especially in the wake of the Manchester Arena inquiry ^5^. Approximately 90,000 CYP under 18 years of age are transported to hospital by ambulance each year for conditions associated with acute pain in England ^6, 7^. Improving the quality of care and experience of this population will have a lasting impact on both their physiological and psychological wellbeing ^8– 10^ and will also reflect on future healthcare encounters.

Evidence suggests few children suffering from acute pain receive analgesic administration from paramedics, ranging from 14–45% ^11– 13^, and approximately 60% do not achieve effective pain relief ^7^ (defined as the abolition or reduction of pain ≥ 2 points on a 10 point scale ^14– 16^). This highlights a significant gap in care. Considering that pain management has been declared a fundamental human right ^17^, urgent action is required to alleviate patient suffering.

Many factors affect pain management processes in the prehospital setting. Pain management is often de-prioritised in emergency care when life-saving interventions for catastrophic haemorrhage, airway management, breathing, and circulation are necessary ^18^. Treating pain after such life-saving interventions are performed is essential. The limited range of analgesic options available to UK ambulance clinicians is in part due to legal restrictions ^19, 20^ which preclude the use of key controlled drugs such as fentanyl by UK registered paramedics, which can be administered intranasally ^21^ or via lozenge ^22^. Nitrous oxide is widely available to UK ambulance clinicians, but it is challenging to administer to CYP due to its cumbersome nature ^23^.

This realist evaluation will further refine the programme theory developed from a previous realist review by collecting and analysing primary qualitative and quantitative data from children and young people, parents and carers, and ambulance clinicians. Consensus workshops will then be used to operationalise the refined programme theory by reviewing and prioritising potential intervention components.

## Methods

### Patient and Public Involvement

A Young Persons Advisory Group (YPAG) was established to advise on the initial design of the improving Pain mAnagement for childreN and young people attendeD by Ambulance (PANDA) Study ^24^. The group was recruited from a UK state-funded secondary school and comprised a total of 25 members. The age of members ranged from 12 to 18 years, 60% were female (n = 15), 64% were White (n = 16), 24% were Asian or Asian British (n = 6), and 13% were of Other or Mixed ethnicity (n = 3). Four of the YPAG members had experience of being in an ambulance with a painful condition, three were in an ambulance for other reasons, and four had witnessed friends or family members going into an ambulance. Members will advise on CYP-facing recruitment materials (leaflets, information sheets) and data collection materials (interview schedules) and will advise on the interpretation of research data (artworks) and findings.

A Parent and Carer Advisory Group (PCAG) was established to advise on the design of the PANDA Study, particularly the parent/carer and CYP facing recruitment and data collection materials. The group consisted of four female parents of children aged 10–18 years. Members will advise on the interpretation of the research findings.

An established patient and public involvement group based at the University of Lincoln (the Healthier Ageing Patient and Public Involvement (HAPPI) group) was involved in the initial design of the PANDA Study. The HAPPI group will continue to be involved in key stages throughout the realist evaluation and consensus workshops, particularly to assist with the interpretation of the findings. The HAPPI Group members will provide input from a “public” perspective and will also bring external expertise to the project from their links to other patient and public involvement groups and from the experience of advising several other prehospital ambulance-based research projects.

### PANDA Study Design

**Figure 1.**
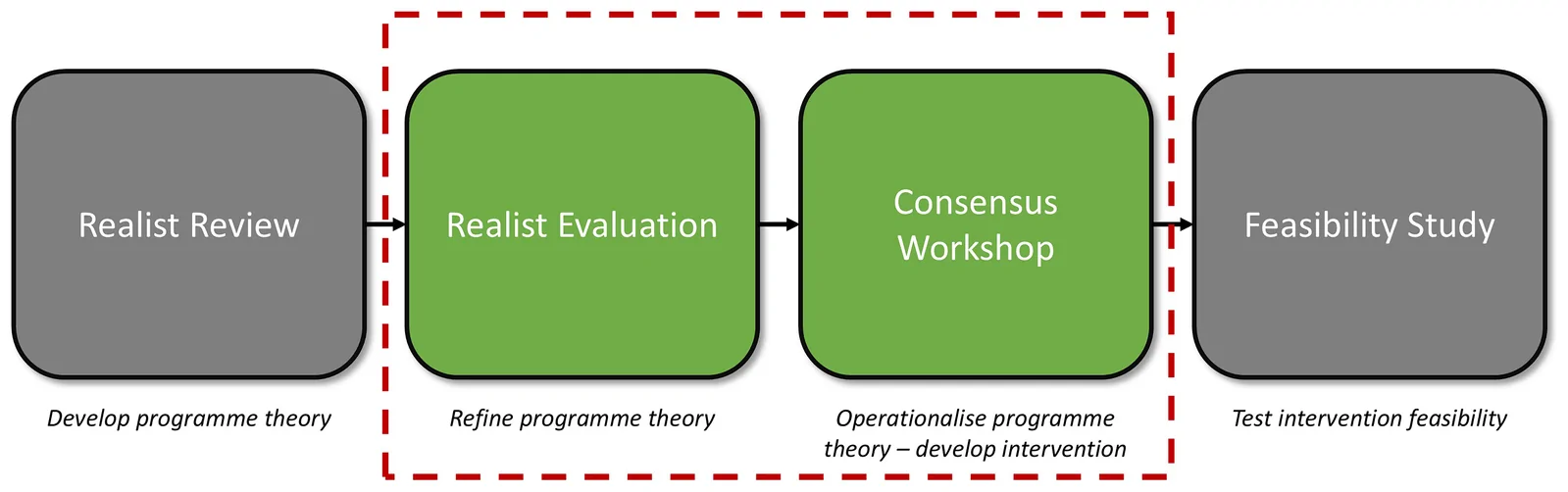
Phase of study. An illustration showing the stage of the study.

The overall PANDA Study is a realist informed complex intervention development and feasibility study, consisting of a realist review, realist evaluation, consensus workshops, and feasibility trial. The aim of the PANDA Study is to develop and test an intervention to improve prehospital pain management for children and young people by exploring what interventions work, for whom, in what context, and how. The protocol for the realist review has been published ^25^. This paper reports the protocol for the realist evaluation and consensus workshops (see Figure 1).

The PANDA Study will be framed within a realist approach, as described by Pawson ^26– 28^, which aligns with the Medical Research Council guidelines for complex intervention development ^29^. A realist approach seeks to understand why, how, to what extent, for whom, and in what circumstances a programme or intervention works ^30^. It assumes that interventions or programmes themselves do not *cause* outcomes; rather, it is the resources offered by the intervention that trigger a response from the participant through underlying unseen **mechanisms** that cause **outcomes** within a specific **context** ^30^. These context-mechanism-outcome configurations (CMOCs) are the foundation on which programme theory is built and may be informed by primary (realist evaluation) or secondary (realist review) data ^27, 28^.

### Realist evaluation

***Study design***

A realist evaluation will be conducted following the guidance of the RAMESES II project ^31^, utilising a multiple case study design ^32^. The multiple case study will utilise interviews, artefacts [arts-based methods: drawings/paintings], diaries, electronic communication methods, and routinely collected clinical data ^32^.

The multiple case study will use three cases, segregated by age:

- *Case 1*: Episodes of acute pain management by ambulance services involving young children aged 0–5 years.
- *Case 2*: Episodes of acute pain management by ambulance services involving children aged 6–11 years.
- *Case 3*: Episodes of acute pain management by ambulance services involving children and young people aged 12–17 years.

The experiences of CYP are likely to vary significantly across the age span, considering that pain is a learnt experience ^33^. There is also known variation in pain management across ages ^7, 34^ therefore, this case segregation is both necessary and justified.

***Setting***

The realist evaluation will be multicentre and will focus recruitment across three NHS ambulance trusts and two NHS children’s emergency departments across England. These sites (East Midlands Ambulance Service NHS Trust, Yorkshire Ambulance Service NHS Trust, North East Ambulance Service NHS Foundation Trust, Sheffield Children's NHS Foundation Trust, Nottingham University Hospitals NHS Trust) serve a population of approximately 12.6 million, covering urban, rural, and coastal areas, with a diverse mix of ethnicities and deprivation ^35– 37^. In addition, social media and website advertising will be used to recruit participants from across England.

***Sample and recruitment***

 The recruitment target is 54 participants: 12 ambulance clinicians, 21 parents/carer, and 21 CYP. This would represent nine CYP and nine parents/carers in Case 1, six CYP and six parents/carers in Case 2, six CYP and six parents/carers in Case 3, and 12 ambulance clinicians that would each provide suitable data for all three cases. This recruitment target was deemed feasible considering time and resource constraints and would provide adequate qualitative data for a meaningful analysis of each case study. More CYP and parents are necessary for Case 1 due to the restriction in eligible age range (4 and 5 years) and the likely brevity of these interviews ^38^.

**Inclusion criteria**

**Children and Young People**:

- aged 4–17 years, inclusive, and
- have recently suffered acute pain in England during the last 12 months, which required an ambulance.

**Parents/carers**:

- whose child or young person under 18 years of age has recently experienced acute pain in England during the last 12 months, which required an ambulance.

**Ambulance clinicians**:

- who have recent experience of managing acute pain in children and young people under 18 years of age in the last 12 months
- has clinical responsibility for the management of children and young people, and
- who are employed by an English NHS ambulance service.

**Exclusion criteria**

**Children and Young People**:

- who have sustained potentially life-threatening injuries or illnesses, such as major trauma (such as limb loss, significant burns >10% of the total body surface area) or cardiac arrest.

**Parents/carers**:

- <16 years of age.

**Recruitment within Ambulance Services**

The identification and recruitment of CYP and parents/carers within ambulance trusts will be led by ambulance clinicians. Paramedics and other ambulance clinicians attending eligible patients will provide standard care, and at the point of hospital handover/discharge on scene, the clinician will hand the parent/carer and child/young person a study recruitment leaflet. The parent/carer and child/young person will then have time to read the information and contact the research team if they wish to find out more information and participate. We anticipate selection bias, where ambulance clinicians may choose to hand leaflets to those whose care was deemed positive. To address this, we will also recruit from children’s emergency departments and online via social media.

**Figure 2.**
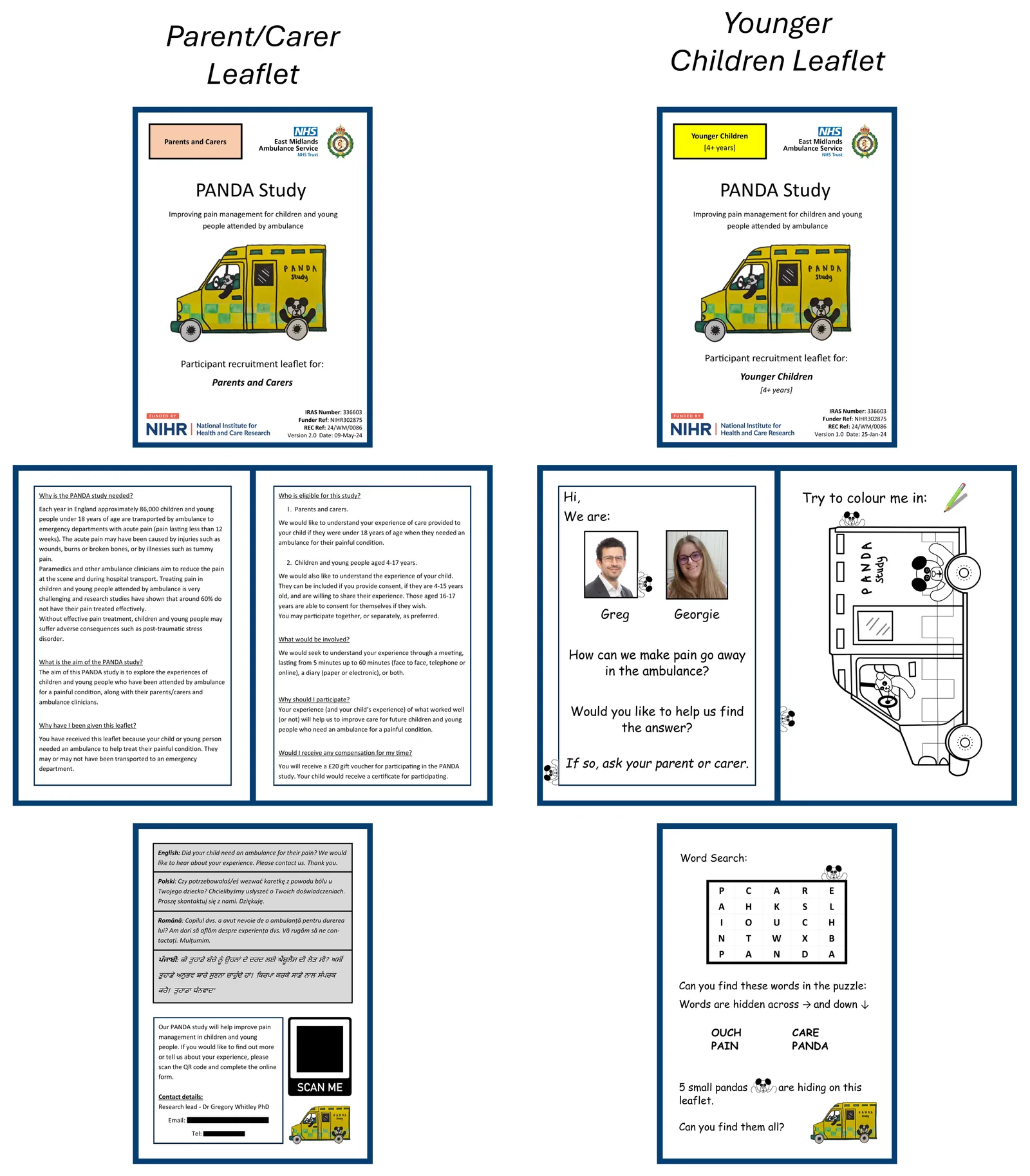
PANDA Study Recruitment Leaflet Examples. Examples of some of the recruitment leaflets used in this study.

Recruitment leaflets were developed (see Figure 2 for examples) with the support of the Young Persons Advisory Group, Parent and Carer Advisory Group, and PANDA Study Working Group.

- A recruitment leaflet labelled “ **Younger Children [4+ years]**” was developed to target children aged approximately 4–9 years. The leaflet contains pictures, a word search, a colouring activity, a “find the hidden pandas” activity, and a small amount of simple information about the PANDA Study.
- A recruitment leaflet labelled “ **Older Children**” was developed to target children aged approximately 10–15 years. The leaflet contains pictures, a word search, and more information about the PANDA Study. The text within this leaflet was developed to a Flesch Reading Ease score of 80 or more (“easy to read”) to maximise readability. Whilst the “Younger Children” leaflet is aimed at those aged 4–9 years, and the “Older Children” leaflet is aimed at those aged 10–15 years, the ambulance clinician or emergency department staff member may use their discretion, along with child/parent/carer choice, to provide the most appropriate leaflet.
- A recruitment leaflet labelled “ **Young People [16–17 years]**” was developed to target young people aged 16 and 17 years. The leaflet is similar to the parent/carer leaflet and contains contact details for the research team. The age range is explicit, as only those aged 16 years or older should possess contact details for the research team.
- A recruitment leaflet labelled “ **Parents & Carers**” was developed to target parents and carers of CYP. The leaflet contains more detailed information on the PANDA Study, along with a scannable QR code and contact details of the PANDA study team where the parent/carer can express an interest in the study and find out more. The text within this leaflet was developed to a Flesch Reading Ease score of 60 or more (“Plain English. Easily understood by 13- to 15-year-old students”) to maximise readability. This leaflet also contains a multilingual statement to encourage the participation of non-English-speaking parents/carers (according to the 2021 census, the three most common non-English languages are Polish, Romanian, and Panjabi; therefore, these three languages are used on the leaflet to encourage non-English-speaking minority populations).

**Figure 3.**
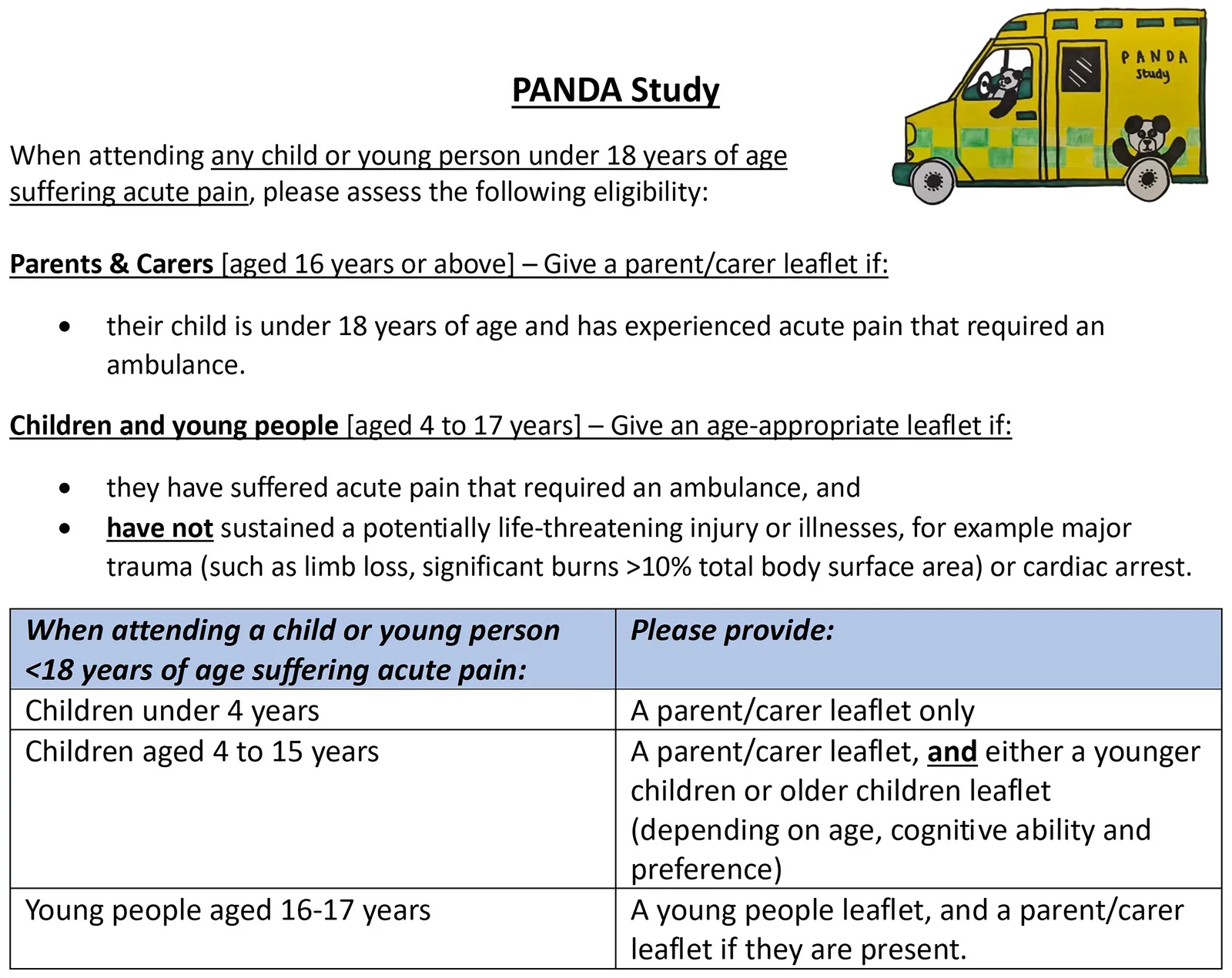
Recruitment Guidance for Ambulance Clinicians. Guidance provided to ambulance clinicians when handing out the study leaflets.

The following guidance will be provided to ambulance clinicians to inform them of which leaflet to provide to whom (see Figure 3).

The identification and recruitment of ambulance clinicians will be facilitated by standard communication channels within the three participating ambulance services, including but not limited to email, staff newsletters, and internal and external social media channels.

**Within children’s emergency department**

The identification and recruitment of CYP and parents/carers within the children’s emergency departments will occur after handover from the ambulance crew. A staff member will screen for eligible participants entering the department by ambulance, who will approach the parent/carer and CYP after initial pain management has occurred. Recruitment leaflets will be handed to the CYP and parents/carers along with an explanation of the study. Parents/carers may express an interest immediately if they wish or take the leaflet away to consider expressing thier interest at a later date. Expressions of interest (EOI) can be made by telephone, email, or by scanning the QR code, which will load a Microsoft Form.

EOIs do not indicate an obligation to participate. Upon receiving an EOI, the research team will share the participant information within five working days, or otherwise as soon as possible, answer any questions, and then discuss the consent and data collection processes once the participant has indicated that they are happy to share their experience.

**Online and social media**

Children and young people, parents and carers, and ambulance clinicians will be recruited across England through website advertising and social media. Given the preferred method of face-to-face data collection for younger children (to enable arts-based methods and the use of toy ambulances), recruitment further afield outside England would have been challenging.

***Data collection***

**Interviews**

Individual semi-structured realist interviews will be conducted with CYP, parents, carers, and ambulance clinicians. Interviews will be audio-recorded and transcribed verbatim. CYP and parents/carers will be interviewed separately, where both parties agree to participate (with the parent/carer in the same room for those aged 4 to 15 years). It was deemed important by our patient and public involvement (PPI) group during study design that data collection occurs separately where possible to ensure that the experience of CYP is gained without the influence of, as far as possible, the parent/carer. However, from a pragmatic perspective, if CYP felt more comfortable with the parent/carer involved in the interview, this would be accommodated. Qualitative data will be anonymised where needed by the lead researcher and research assistant. For example, if a parent/carer names their child or young person during an interview, this would be redacted.

**Arts-based materials**

Arts-based methods (Lego/toy cars/ambulances/paper/card/pens/felt tips) will be used during face-to-face interviews, particularly for younger children, to facilitate the communication of their experiences. These artefacts will be collected along with field notes taken by the researcher during the interviews. This will follow methods previously used to explore experiences of children aged 4–8 years suffering acute pain in the emergency department ^38^. Interviews will be performed by the lead researcher and the research assistant face-to-face at a location to suit the participant, online via videoconference, or telephone, depending on participant preference and social restrictions.

**Diaries**

Diaries will be used by CYP where preferred, and by parents, to document conversations with their child or young person, along with any observed behavioural changes. Diaries will be electronic (Microsoft Forms) or paper according to participant preference.

**Electronic communication**

Electronic communication will be used by young people (12–17 years) where preferred, such as a secure messaging service (Microsoft Teams chat, within a unique channel), to explore their experience. Microsoft Teams chats would be scheduled for time slots of up to 60 minutes and conducted with the parent/carer present for those aged 12–15 years.

**Routinely collected clinical record data**

Anonymised routinely collected electronic clinical record data will be collected from two ambulance services (East Midlands Ambulance Service NHS Trust and Yorkshire Ambulance Service NHS Trust) for CYP aged under 18 years, who had a Glasgow Coma Scale score of 15 documented, or an AVPU (alert, responsive to voice, responsive to pain, unresponsive) of “alert” documented, who had a clinical impression suggestive of acute pain (such as soft tissue injury, thermal injury, abdominal pain, etc.) for a 3-year period (01-Jan-2021 to 31-Dec-2023). The extracted data will include the following:

- Incident date and job times (call received, resource mobile, arrival at scene, departure from scene, hospital arrival, handover, and clear)
- Patient age, sex, and ethnicity
- Location type (home, public, school, where available)
- Index of multiple deprivation (generated from incident & home postcode)
- Source of pain (chief complaint and clinical impression(s))
- Pain scores and times (all documented pain scores, times and scales used)
- Treatments and times (pharmacological and non-pharmacological, and times)
- Outcome (Treated and transported / treated and discharged)
- Ambulance clinician data, such as the highest rank clinician on scene (doctor/paramedic/technician), experience, and sex of the clinicians, where available.

Clinical record data will be de-identified by both ambulance services prior to their transfer to the University of Lincoln servers for analysis. All data will be stored on the University of Lincoln secure Microsoft OneDrive and SharePoint drives, accessed through password-protected University of Lincoln computers.

***Data analysis***

**Figure 4.**
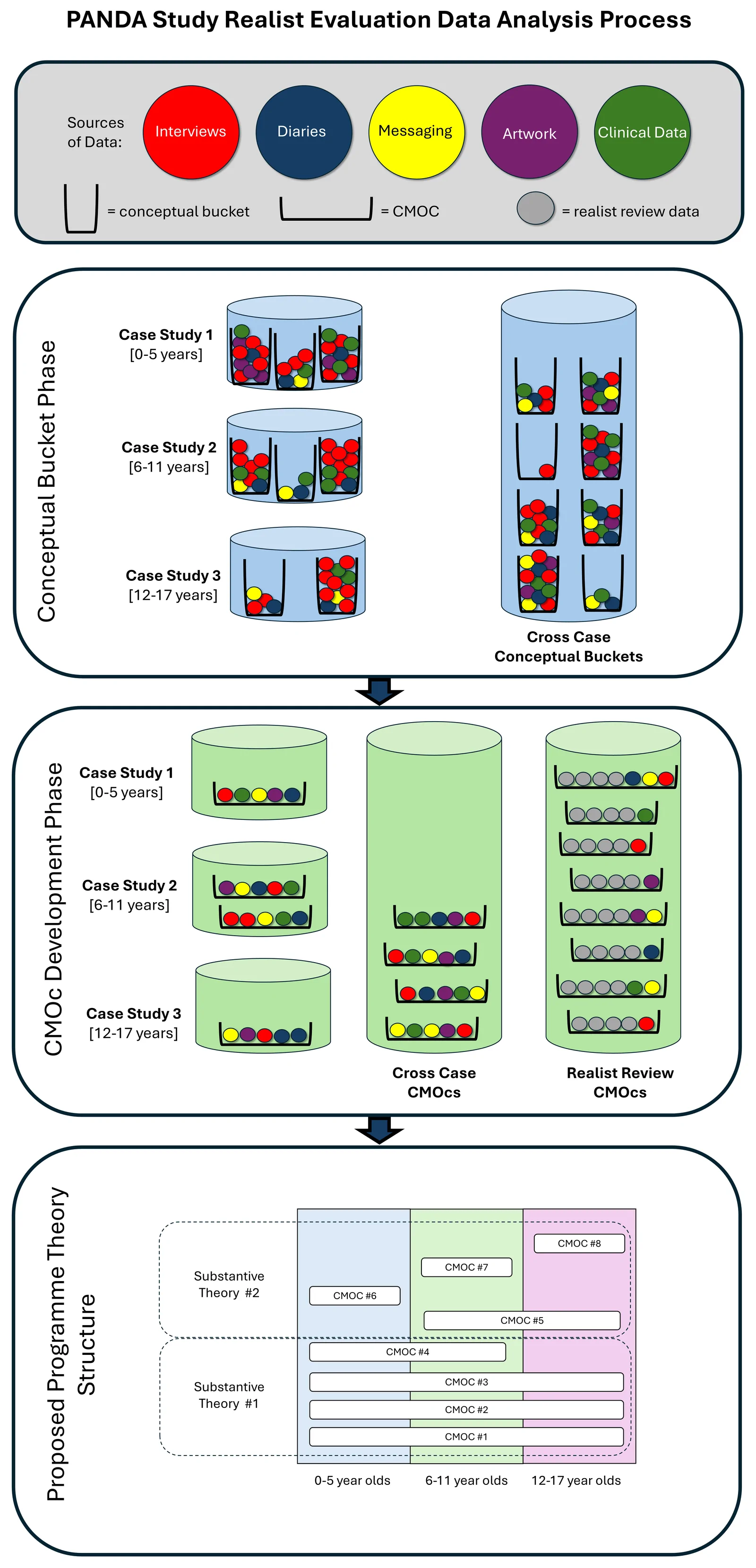
Realist Evaluation Data Analysis Process. An illustration of the realist evaluation data analysis process. CMOC – Context-mechanism-outcome configuration.

The analysis process within this realist evaluation will be highly complex, conforming to the principles of multiple case study analysis and realist logic of analysis (see Figure 4 for the realist evaluation data analysis process.

Qualitative data will be analysed in line with a realist logic of analysis within each case to facilitate the development of CMOCs and refinement of the PT developed from the realist review. This process of programme theory refinement will follow methods previously described by Dalkin *et al*. ^39^, using QRS NVivo software. Recorded audio files will be stored on the University of Lincoln secure Microsoft servers and transcribed verbatim by the research team or professional services, as required. The transcripts will be checked by the lead researcher and research assistant prior to data analysis. Transcripts will be combined with field notes and analysed within QRS NVivo, along with data collected from diaries and electronic communication.

Arts-based materials will be analysed by exploring the denoted content (what is presented in the art?) along with the connoted content (what are the underlying intentions/meanings of the presented imagery?) with the latter using a semiotic approach ^40^. Analytic interpretations of the artefacts will be documented by the research team within a Microsoft Word document and incorporated into the NVivo file for analysis.

Descriptive statistics along with multivariable regression analyses will be conducted on the anonymised clinical data to help identify predictors of effective pain relief, similar to previous work ^7^. These data are necessary to help inform the “outcome” aspect of the CMO configurations, of which pain score is deemed an important outcome. The quantitative analysis will be segregated by case. To incorporate quantitative data into the realist logic of analysis, a narrative description of the quantitative results will be produced and uploaded into NVivo for coding.

**Conceptual bucket phase**

Data from interviews, diaries, electronic messaging, arts-based materials, and clinical data will initially be coded into “conceptual buckets” within cases and across cases. It is expected that all qualitative data from children and young people will be coded into their respective case. Most of the qualitative data from parents/carers will be coded into their child’s respective case, except when referring to an older/younger sibling for example. Only some of the data from ambulance clinicians will be coded into the respective case for the incident they discuss, with the remainder coded into the other two cases or the cross-case section. We will use the existing conceptual buckets created within our previous realist review and create new ones as needed. This will allow us to group data on similar concepts or themes.

**CMOC development phase**

After coding is complete, for each conceptual bucket, we will determine what may be functioning as contexts, what may be functioning as outcomes, and what may be functioning as mechanisms. Through an iterative process, new CMOCs will be developed. Where data further substantiates previously developed CMOCs from the realist review, these data will be coded to the existing CMOCs. Where data contradicts previously identified CMOCs from the realist review, these will be coded, and the CMOCs revised or removed after discussion with the PANDA Study working group and other stakeholder groups.

CMOCs will be developed within and across cases, as shown in Figure 4. Once CMOC generation is complete, we will assess across cases to see if any can be combined. For example, a CMOC within Cases 1 and 2 may be combined, creating a CMOC in the context of 0–11 year old children.

There may be instances where data coded from documents contradict each other or only supply a part of the CMOC. We may juxtapose, reconcile, adjudicate, consolidate or situate ^27^ findings throughout the analytic process, as necessary.

Once the previously identified CMOCs from the realist review have been further substantiated, revised, or removed, and all new CMOCs have been developed, the programme theory will be revised.

**Programme theory revision**

A process of iterative consultation of the PT with the PANDA Working Group, PANDA Ambulance Clinician Advisory Group, and the PANDA Young Persons Advisory Group will occur throughout the analysis to ensure that the theory is well grounded and acceptable to key stakeholders.

CMOCs developed during the previous realist review were not created according to age (as will be the case for new CMOCs developed during this realist evaluation). Therefore, we will perform an iterative process of review for each CMOC that is carried forward from the realist review to determine as far as possible how the CMOC relates to age. Suitable substantive theories will then be identified and used to support the final PT.

**Software**

QRS NVivo version 14 (copyright licence obtained) will be used to facilitate the realist analysis. Stata version 18 (copyright licence obtained) will be used to analyse the clinical record data.

***Ethical considerations***

Favourable research ethics committee approval was received for this realist evaluation, written informed parental consent will be obtained for all participants aged 4–15 years and written informed consent will be obtained from participants aged 16 years and above. Please see Ethical Considerations section later for more details.

### Consensus workshops

The PT developed from the realist evaluation will be mapped to the Behaviour Change Wheel ^41^ to identify behaviour change techniques ^42^ that could be incorporated into an intervention to improve prehospital pain management for children and young people. For example, the intervention will likely involve an educational component aimed at frontline ambulance clinicians. Behaviour change intervention component development will be an iterative process of discussion and revision involving the PANDA Working Group, YPAG, parent and carer advisory group (PCAG) and ambulance clinician advisory group (ACAG). Once a list of viable components has been drafted, the first consensus workshop will be conducted.

***Study design***

Two consensus workshops will be conducted ^43^, utilising the modified nominal group technique ^44^ to review, generate, and prioritise the intervention components. It is important to conduct workshops separately for ambulance staff and for children, young people, parents, carers, and members of the public, as their level of understanding is likely to require separate, tailored approaches to maximise engagement and ensure meaningful participation.

**Workshop #1** will involve parents/carers, older children, and public contributors, who will review the PANDA Study findings to date, review a draft list of potential intervention components that have been mapped to the Behaviour Change Wheel, have the opportunity to generate additional intervention components, and vote to prioritise components by perceived importance. Voting will be online using a Likert scale of importance for each component.

**Workshop #2** will involve ambulance service clinicians, quality leads, and educators, who will review the PANDA Study findings, review a draft list of potential intervention components (revised from Workshop #1), have the opportunity to generate additional intervention components, and prioritise the list of potential intervention components. Voting will be online using a Likert scale of importance for each component.

Upon completion of both consensus workshops, prioritisation data will be combined to produce a list of the most important intervention components. For example, this may be the top five ranked components from each workshop. These components will then be drafted into the intervention, utilising an iterative approach of review and refinement while engaging with key stakeholder groups (YPAG, ACAG, PCAG, and HAPPI Group) and the PANDA Study Working Group.

***Setting***

Workshops will be held at the University of Lincoln, which has good public transport routes and visitor parking. All the buildings are easily accessible with ramps and lifts available. Participant travel costs will be covered along with compensation for CYP, parents, carers, and public representatives in line with NIHR INVOLVE rates. The events will be hybrid, face-to-face, and online, facilitating the involvement of those unable to travel.

***Sample and recruitment***

Children, young people, parents/carers, and ambulance clinicians who participated in the realist evaluation will be invited to express an interest to participate in the consensus workshops, and will be selected purposively to ensure that a mix of age and sex is achieved. We will also recruit ambulance service clinicians and quality and education leaders from across England. We aim to recruit between 8–20 participants per workshop.

**Inclusion criteria**

**Children and Young People**:

- aged 8–17 years, inclusive, and
- have recently suffered acute pain in England during the last 18 months, which required an ambulance.

**Parents/carers**:

- whose child or young person under 18 years of age has recently experienced acute pain in England during the last 18 months, which required an ambulance.

**Ambulance clinicians**:

- who have recent experience of managing acute pain in children and young people under 18 years of age in the last 12 months
- has clinical responsibility for the management of children and young people, and
- who are employed by an English NHS ambulance service.

**Ambulance quality and education leaders**:

- who are employed by an English NHS ambulance service.

**Members of the YPAG and Healthier Ageing Patient and Public Involvement (HAPPI) group**

**Exclusion criteria**

**Children and Young People**:

- who have sustained potentially life-threatening injuries or illnesses, such as major trauma (such as limb loss, significant burns >10% of the total body surface area) or cardiac arrest.

**Parents/carers**:

- <16 years of age.

***Data collection***

Consensus workshops will run for up to six hours, including a large break for lunch and several comfort breaks. Workshops will be audio-recorded, with detailed minutes and appropriate actions drafted. Table 1 shows the proposed workshop structure.

**Table 1.** Proposed Consensus Workshop Agenda.

Activity	Groups	Time
Welcome and Introductions	Whole Group	30 minutes
Presentation of the PANDA Study findings	Whole Group	30 minutes
**Comfort Break**		**30 minutes**
Review of intervention components	Small Groups	30 minutes
Round-robin generation of new intervention components	Small Groups	30 minutes
**Comfort Break**		**30 minutes**
Member Voting Round #1	Whole Group	20 minutes
**Lunch**		**60 minutes**
Review/discussion of results	Whole Group	20 minutes
Member Voting Round #2	Whole Group	20 minutes
**Comfort Break**		**30 minutes**
Feedback and final discussion	Whole Group	30 minutes
**Total**		**6 hours**

The lead researcher and research assistant will take field notes during the consensus workshop discussions. These will be collated, summarised, and reviewed by the PANDA Working Group with the onward actions discussed.

Electronic data for the nominal group technique used for the consensus workshops will be collected and analysed in real time. The results will be collated and illustrated to the group using appropriate charts and tables. A further round of electronic data will then be collected, analysed, and reported back to the group at the end of the workshop.

***Data analysis***

Utilising the nominal group technique ^44^, member online voting will take place within a consensus workshop involving a range of key stakeholders. The aim will be to determine the highest-priority intervention components through consensus. There is no clear formula or definition of consensus for use in modified nominal group technique studies, with previous studies utilising a variety of approaches, primarily focusing on prioritisation rather than consensus ^43, 45, 46^. Defining consensus a priori is challenging, and assigning an arbitrary cut-off point for consensus is unhelpful. Instead, we will focus on the prioritisation of components along with the qualitative data and rates of agreement, which will provide the justification for prioritisation.

Electronic data will be analysed using MS Excel, where mean scores and proportions will be calculated for the intervention components. Qualitative data collected from field notes and transcribed audio recordings will be analysed using QRS NVivo version 14.

High-priority intervention components from Workshops 1 and 2 will be combined and used to inform intervention development.

### Ethical considerations

***Approvals***

A favourable ethical opinion was obtained from the West Midlands – Coventry and Warwickshire Research Ethics Committee [24/WM/0086], and NHS Health Research Authority approval was obtained [Integrated Research Application System ID: 336603].

***Consent***

Written informed parental consent will be obtained for all participants aged 4–15 years and written informed consent will be obtained from participants aged 16 years and above.

Children and Young People aged 4 to 15 years, inclusive.

There is no statute in England, Wales, or Northern Ireland governing a child's right to consent to participate in research other than a Clinical Trial of an Investigational Medicinal Product (CTIMP) ^47^. Therefore, the approach taken to consent CYP aged 4 to 15 years (inclusive) within the PANDA study will be guided by Health Research Authority guidance ^47^ along with YPAG, CPAG, and PANDA Working Group input.

CYP aged 4 to 15 years inclusive:

- Will be provided information about the PANDA study in the form of an age-appropriate recruitment leaflet and participant information sheet.
- Will have their questions answered and their wishes addressed appropriately.
- Will be able to provide verbal informed assent where appropriate and, where able, sign an age-appropriate informed assent form prior to participation in WP2a and WP2b.
- Must have parental/carer written informed consent provided prior to involvement in WP2a or WP2b.

Young people aged 16 and 17 years.

Young people aged 16 and 17 years are presumed to be capable of giving consent on their own to participate in Clinical Trials of Investigational Medicinal Products (CTIMPs) ^47^. Therefore, it is reasonable to assume that if those aged 16 and 17 years can provide consent for CTIMPS, and common law presumes that young people aged 16 and 17 years are usually competent to give consent to treatment, then 16- and 17-year-old participants are able to provide full informed consent for non-CTIMP research studies, such as the PANDA study.

Young people aged 16 and 17 years:

- Will be provided information about the PANDA study in the form of a recruitment leaflet and participant information sheet.
- Will have their questions answered and their wishes addressed appropriately.
- Will have their capacity assessed.
- Must provide written informed consent prior to participation in WP2a or WP2b.
- May participate without parent/carer approval; however, parent/carer approval will be encouraged.

Adults aged 18 years and over.

- Will be provided information about the PANDA study in the form of a recruitment leaflet and participant information sheet.
- Will have their questions answered and their wishes addressed appropriately.
- Will have their capacity assessed.
- Must provide written informed consent prior to participation in WP2a or WP2b.

Capacity assessment

Although mental capacity should be assumed in all adults, for the purpose of this research and to obtain consent from participants aged 16 years and above, the PANDA research team will ensure that participants:

- understand the purpose and nature of the research,
- understand what the research involves, its benefits (or lack of benefits), risks, and burdens,
- understand the alternatives to taking part,
- can retain the information long enough to make an effective decision,
- can make a free choice,
- can make this particular decision at the time it needs to be made (though their capacity may fluctuate, and they may be capable of making some decisions but not others depending on their complexity).

This will be achieved during responses to expressions of interest, discussions arising between the expression of interest and consent, during the consent process, and prior to involvement in WP2a and WP2b. Potential participants aged ≥ 16 years who lack mental capacity will not be eligible for the study.

For those aged 4–15 years (inclusive), the ability to communicate and engage in the research process will substitute the capacity assessment, with full support of parental/carer informed consent.

## Stakeholder involvement

In addition to the patient and public involvement described above, a variety of key stakeholder groups were developed to advise the PANDA Study and provide oversight.

### Ambulance Clinician Involvement

An Ambulance Clinician Advisory Group (ACAG) has been established with expertise from the fields of clinical practice, education, and senior leadership. The group will meet at several stages of the study to provide insights and expert knowledge to help with the development and refinement of ambulance clinician-facing documents and to facilitate the development and refinement of the programme theory.

### PANDA Study Working Group

A bespoke PANDA Study Working Group was established to provide guidance and oversight of the full PANDA Study. The group consists of academics with expertise in realist methods, the prehospital setting and the population of children and young people, clinicians, ambulance service representatives, and clinical and non-clinical psychologists. The group will meet monthly or more, if required, to discuss the progress of the study and provide expertise at all stages.

### PANDA Study Steering Committee

An international steering group containing experts from the fields of paediatric emergency care, prehospital interventional research methods, prehospital qualitative research methods, and a public representative was established to provide external oversight to the PANDA Study. The groups will meet every six months, or more if needed, throughout the PANDA Study.

## Data Availability

No data are currently available, as this is a study protocol. Upon completion, according to our Data Access Management Plan https://fundingawards.nihr.ac.uk/award/NIHR302875 and our ethical approval, we will be unable to share the primary data from this research in an open access repository due to the potential to identify participants. However, key de-identified data used to inform the analysis will be made available in a Supplementary File. In line with our Data Access Management Plan, research data may be available upon reasonable request, and we will handle requests for research data on a one to one basis. Such requests should be made by email to: researchteam@emas.nhs.uk

## Software availability

NVivo 14 is proprietary software, and free alternatives such as QualCoder ( https://qualcoder.wordpress.com/) could be used. Stata 18 is proprietary software, and free alternatives such as R ( https://www.r-project.org/) could be used.
