# Supplementary material for: Improving Pain management for children and young people attended by Ambulance (PANDA): protocol for a realist evaluation and consensus workshops
Source: NIHR Open Res. 2026 Aug 13;5:88. Originally published 2025 Oct 1. [Version 3] doi: 10.3310/nihropenres.14025.3 (PMC13554774; doi:10.3310/nihropenres.14025.3)
Supplement: Peer review history

# Reviewer report: Reviewer response for version 3

**DOI:** 10.3310/nihropenres.15738.r41623

**Published:** 2026-08-26

**Authors:** Daniela Ghio, Danielle Mountain

**Affiliations:** 1 The University of Manchester, Manchester, England, UK; 2 Edge Hill University Faculty of Health Social Care and Medicine (Ringgold ID: 156525), Ormskirk, England, UK

Thank you for your thorough and thoughtful responses to the reviewers' comments. I appreciate the care taken to address all points raised, which has strengthened the protocol considerably. I have no further comments and look forward to seeing this important and timely research progress.

Is the study design appropriate for the research question?

Yes

Is the rationale for, and objectives of, the study clearly described?

Partly

Are sufficient details of the methods provided to allow replication by others?

Partly

Are the datasets clearly presented in a useable and accessible format?

Not applicable

Reviewer Expertise:

Child health literacy; health psychology; paediatric pain; medication adherence

We confirm that we have read this submission and believe that we have an appropriate level of expertise to confirm that it is of an acceptable scientific standard.

# Reviewer report: Reviewer response for version 2

**DOI:** 10.3310/nihropenres.15656.r40850

**Published:** 2026-07-22

**Authors:** Daniela Ghio, Danielle Mountain

**Affiliations:** 1 Edge Hill University Faculty of Health Social Care and Medicine (Ringgold ID: 156525), Ormskirk, England, UK; 2 The University of Manchester Division of Psychology and Mental Health (Ringgold ID: 171151), Manchester, England, UK

This is an interesting, well-developed project addressing an important topic, using appropriate approaches to address the aim. Thank you for the opportunity to provide a review. There is clear evidence throughout of the careful processes taken to consider and improve inclusivity.

 We have some suggestions to improve clarity and replicability.

1. In the introduction, although implied in the preceding text, it would be useful to explicitly state within the aim that the programme theory intends to inform interventions to improve pre-hospital pain management for children and young people.
2. The potential for selection bias through ambulance clinicians selectively offering recruitment materials is considered and alternative recruitment avenues described. However, can you provide further details on how you will monitor, assess or minimise selection bias? For example, will you monitor recruitment from different sources to identify whether particular routes result in an over-representation of participants, and will strategies be used to address this potential bias?
3. The protocol describes the qualitative methods and purposes of the interviews, diaries, activities and electronic communication. However, it would be beneficial to provide the general topics of questions that will be explored in each component (or reference supplementary materials if available). 
4. The use of diaries is discussed to document conversations and behavioural changes however there is limited detail regarding their format and structure. Providing these details will improve transparency and replication, for example will participants be provided with structured prompts, examples of behaviours to record, a check list or open-ended questions?
5. The protocol states that routine clinical data will be collected from two NHS ambulance services, while recruitment for the wider study involves three services. Please clarify the rationale for collecting data from only two services. Also, please discuss the potential implications of this (e.g. do these capture a range of ambulance service contexts and practices).
6. Can the authors provide more detail on how they will promote diversity within recruitment and what specific strategies will be used to engage participants from a range of backgrounds, experiences, and demographic groups (perhaps clarifying if the different types of data collection is to address this)?
7. A developmental approach is valuable when designing research with children and young people; however, chronological age should be viewed as a guide rather than a strict criterion. Given the substantial variation in development, experience, and preference within age groups, allowing overlap between age bands can provide greater flexibility and help ensure that participation is tailored to the needs of individual children and young people. 
  Allowing overlap between age groups also creates opportunities for children and young people to engage in activities that best match their preferences, communication styles, and developmental needs, rather than being constrained by chronological age alone. If keeping with the specific ages it would be recommended to make the justification clear on relying on these distinct categories. 

Is the study design appropriate for the research question?

Yes

Is the rationale for, and objectives of, the study clearly described?

Partly

Are sufficient details of the methods provided to allow replication by others?

Partly

Are the datasets clearly presented in a useable and accessible format?

Not applicable

Reviewer Expertise:

Child health literacy; health psychology; paediatric pain; medication adherence

We confirm that we have read this submission and believe that we have an appropriate level of expertise to confirm that it is of an acceptable scientific standard, however we have significant reservations, as outlined above.

# Reviewer report: Reviewer response for version 2

**DOI:** 10.3310/nihropenres.15656.r40845

**Published:** 2026-07-20

**Authors:** Fareha Nishat

**Affiliations:** 1 The Hospital for Sick Children, Toronto, Ontario, Canada

Thank you for the opportunity to review this protocol. This article describes the realist evaluation and consensus working group phases of the PANDA project. Overall, this is a comprehensive and well-designed study that aims to identify realistic and feasible interventions to improve pain management for children and young people who use ambulance services. The methods are thoughtfully developed and clearly presented. I also commend the research team for their intentional and meaningful engagement of patients, families, and other relevant stakeholders through advisory groups to inform the study design. I have a few suggestions that may help strengthen the rationale and clarify certain aspects of the study procedures.

 Introduction

 - The introduction provides a helpful overview of the scope of the problem; however, the rationale for the study could be more explicit. It may be useful to include one or two sentences clearly outlining the implications of the identified evidence or practice gap and explaining why the proposed research is needed. For example, the authors could briefly clarify how the current limitations in pain management necessitate evidence on which interventions may be feasible and effective within ambulance services.

 - The introduction would also benefit from a clearer statement of the study objectives or aims. Although these are described in greater detail in the PANDA study design section on page 5, including a concise version in the final paragraph of the introduction would help connect the study rationale to the proposed methods.

 Methods

 - The methods for both the realist evaluation and consensus working group phases are comprehensive and well described. The authors provide helpful explanations for the methodological decisions made throughout the study.

 - The intended purpose of the diaries and electronic communications was somewhat unclear. It would be helpful to explain what information these methods are intended to capture and how they will complement the interviews or other data sources.

 - It was also unclear whether individuals who participate in the realist evaluation may subsequently participate in the consensus workshop. The authors may wish to clarify whether participation in both phases will be permitted. If there is a concern that prior participation could influence the consensus process, the authors could consider including a exclusion criterion for the consensus workshop, or explain how any potential overlap will be managed.

Is the study design appropriate for the research question?

Yes

Is the rationale for, and objectives of, the study clearly described?

Partly

Are sufficient details of the methods provided to allow replication by others?

Yes

Are the datasets clearly presented in a useable and accessible format?

Not applicable

Reviewer Expertise:

My primary methodological expertise is in epidemiology and quantitative health research, across several disease domains including pediatric chronic pain. But I also have experience conducting and publishing qualitative, patient and family-engaged research in pediatric settings. This experience is relevant to my assessment of the current protocols participant engagement and qualitative data-collection procedures. Although realist evaluation is not my primary area of methodological expertise, I have reviewed the protocol from the perspectives of study design, clarity, feasibility, pediatric health research, and the integration of qualitative and quantitative methods.

I confirm that I have read this submission and believe that I have an appropriate level of expertise to confirm that it is of an acceptable scientific standard.

# Reviewer report: Reviewer response for version 1

**DOI:** 10.3310/nihropenres.15253.r37997

**Published:** 2025-12-11

**Authors:** Mary Ani–Amponsah

**Affiliations:** 1 University of Ghana, Accra, Ghana

A very interesting study with relevant details aimed at improving paediatric pain management, typically in emergency transport situations. This aspect of care is a neglected area in most childcare giving settings; the authors are commended for the extensive work done.  The realist approach is appropriate for enabling us to understand the what, the why, how, to what extent, for whom, and in what situations pain interventions work within specific contexts.

 However, to provide a holistic approach to addressing paediatric pain in contemporary practice, it is important that the authors include the evidence on pain scales for assessing newborns, infants, toddlers; the manuscript does not provide adequate information on this younger population.

 What are the anticipated cultural variations assessors must consider prior to delivering  pain interventions, and how does that impact quality care delivery?

Is the study design appropriate for the research question?

Yes

Is the rationale for, and objectives of, the study clearly described?

Yes

Are sufficient details of the methods provided to allow replication by others?

Yes

Are the datasets clearly presented in a useable and accessible format?

Yes

Reviewer Expertise:

Maternal and Newborn Health

I confirm that I have read this submission and believe that I have an appropriate level of expertise to confirm that it is of an acceptable scientific standard, however I have significant reservations, as outlined above.

# Response

**Published:** 2026-05-28

**Authors:** Gregory Whitley

**Affiliations:** Clinical Audit and Research Un, East Midlands Ambulance Service NHS Trust, Lincoln, UK

Thank you for reviewing our study protocol and for the positive comments. You describe the need to include evidence on pain scales for assessing newborns, infants, toddlers. This information is pertinent to the Data Collection section of the Realist Evaluation, therefore we have added a sentence to describe standard UK ambulance service pain assessment tools used for children and young people. You also describe the need to identify any cultural variations among those assessing pain and its impact on quality of care. We agree this is an important consideration. Whilst the identification of any cultural differences and any implicit bias found within this study will be left to the reporting of the study findings, we have added a sentence to the Introduction of this protocol to explain that cultural differences and implicit bias likely influence quality of care. Thank you for your review and feedback, we believe the manuscript is now much improved.
